# Arsenic Induces p62 Expression to Form a Positive Feedback Loop with Nrf2 in Human Epidermal Keratinocytes: Implications for Preventing Arsenic-Induced Skin Cancer

**DOI:** 10.3390/molecules22020194

**Published:** 2017-01-24

**Authors:** Palak Shah, Elaine Trinh, Lei Qiang, Lishi Xie, Wen-Yang Hu, Gail S. Prins, Jingbo Pi, Yu-Ying He

**Affiliations:** 1 Department of Medicine, Section of Dermatology, University of Chicago, Chicago, IL 60637, USA; palaks@uchicago.edu (P.S.); lqiang@cpu.edu.cn (L.Q.); 2 Committee on Molecular Pathogenesis and Molecular Medicine, University of Chicago, Chicago, IL 60637, USA; 3 Department of Biological Sciences and Department of Chemistry, University of Illinois at Chicago, Chicago, IL 60607, USA; etrinh3@uic.edu; 4 Department of Urology, College of Medicine, and University of Illinois Cancer Center, University of Illinois at Chicago, Chicago, IL 60612, USA; lishixie@uic.edu (L.X.); wyhu@uic.edu (W.-Y.H.); gprins@uic.edu (G.S.P.); 5 Program of Environmental Toxicology, School of Public Health, China Medical University, Shenyang 110122, China; jbpi@cmu.edu.cn

**Keywords:** arsenic, p62, Nrf2, keratinocytes, apoptosis, proliferation

## Abstract

Exposure to inorganic arsenic in contaminated drinking water poses an environmental public health threat for hundreds of millions of people in the US and around the world. Arsenic is a known carcinogen for skin cancer. However, the mechanism by which arsenic induces skin cancer remains poorly understood. Here, we have shown that arsenic induces p62 expression in an autophagy-independent manner in human HaCaT keratinocytes. In mouse skin, chronic arsenic exposure through drinking water increases p62 protein levels in the epidermis. Nrf2 is required for basal and arsenic-induced p62 up-regulation. p62 knockdown reduces arsenic-induced Nrf2 activity, and induces sustained p21 up-regulation. p62 induction is associated with increased proliferation in mouse epidermis. p62 knockdown had little effect on arsenic-induced apoptosis, while it decreased cell proliferation following arsenic treatment. Our findings indicate that arsenic induces p62 expression to regulate the Nrf2 pathway in human keratinocytes and suggest that targeting p62 may help prevent arsenic-induced skin cancer.

## 1. Introduction

Exposure to inorganic arsenic in contaminated drinking water poses an environmental public health threat for hundreds of millions of people in the US and in the world, including Taiwan, Mexico, Mongolia, Argentina, India, and Bangladesh [1]. Epidemiological studies have demonstrated a strong association of high levels of arsenic exposure with elevated risk of many human diseases, including skin cancer [2].

Arsenic is known as an endocrine disruptor and a human carcinogen. In humans, arsenic is known to cause cancers in the skin as well as in internal organs [2]. Arsenic exposure causes skin abnormalities including hyperpigmentation (melanosis) and hyperkeratosis (keratosis), which are early signs of arsenic poisoning. These lesions are considered the precursors of arsenic-induced skin cancers [3]. Arsenic synergizes with sunlight exposure, smoking, and occupational exposures in increasing the risk of skin lesions in Bangladesh [4]. In the Tg.AC mouse model, which expresses activated v-Ha-ras transgene, when offspring receive topical 12-*O*-tetradecanoyl phorbol-13-acetate (TPA) through adulthood, adult or fetal arsenic exposure increases skin tumorigenesis sensitivity [5,6]. Arsenic exposure through drinking water increases skin tumorigenesis induced by UV radiation [7]. In all the mouse models, arsenic alone is not carcinogenic [5,6,7]. These findings suggest that arsenic acts as a co-carcinogen in the skin in mice. In immortalized non-tumorigenic human HaCaT keratinocytes, environmentally relevant arsenic exposure causes malignant transformation in association with increased activation of the transcription factor Nrf2 [8]. It is possible that arsenic modulates multiple signaling pathways to facilitate oncogenic processes [2,9].

One of the signaling pathways in carcinogenesis can be p62, also known as Sequestosome 1 (SQSTM1). p62 is a multidomain protein that interacts with cargos for autophagic degradation as well as several key signaling components [10]. Macroautophagy (hereafter autophagy) is a catabolic process by which unwanted or damaged cellular proteins, cytoplasm, and organelles are captured and targeted for proteolytic degradation in lysosomes [11,12]. p62 has been shown to be both a selective autophagy substrate and an autophagy adaptor protein, and is known to promote tumorigenesis [10,13,14,15]. It is found to be up-regulated in several human cancers, including lung cancer, breast cancer, and melanoma [16,17,18,19]. Furthermore, the oncogenic Ras pathway induces p62 expression to activate the NF-κB pathway and drive lung tumorigenesis [20]. Our recent work and preliminary studies have shown that p62 is up-regulated in human skin tumors [21], and that p62 promotes cell proliferation and migration by stabilizing the oncogenic transcription factor Twist1 [21]. Twist1 is a core regulator in both early embryonic morphogenesis and tumor metastasis [22]. It induces loss of E-cadherin-mediated cell-cell adhesion and promotes the epithelial-mesenchymal transition (EMT) [23]. Indeed, a recent report has shown that arsenic inhibits autophagic flux, results in p62 accumulation, and thus activates Nrf2 through a p62-dependent mechanism [24]. It is possible that p62 up-regulation plays an important role in arsenic carcinogenicity.

Here we have shown that in mouse skin, chronic arsenic exposure through drinking water increases p62 protein levels in the epidermis. Arsenic induces p62 expression through Nrf2 in an autophagy-independent manner in human HaCaT keratinocytes. p62 knockdown reduced arsenic-induced Nrf2 activity, induced sustained p21 up-regulation, and decreased cell proliferation. p62 induction is associated with increased proliferation in the mouse epidermis. Our findings suggest an important role of p62 in arsenic-induced skin tumorigenesis and that targeting p62 may help prevent arsenic-induced skin cancer.

## 2. Results

### 2.1. Arsenic Induces p62 Up-Regulation In Vitro and In Vivo

**Figure 1:**
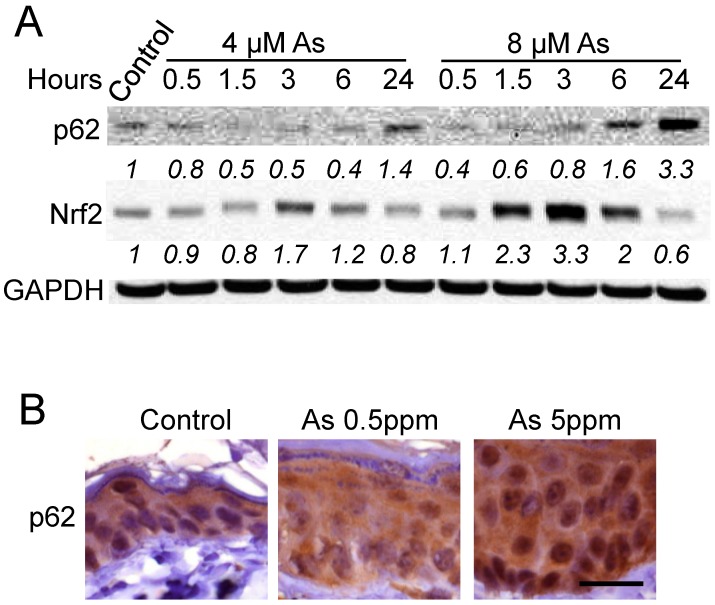
Arsenic induces p62 up-regulation in vitro and in vivo: (**A**) immunoblot analysis of p62, Nrf2 and GAPDH in HaCaT cells treated with vehicle control or arsenic (4 or 8 μM) over a time course; and (**B**) immunohistochemical analysis of p62 in the skin of nude mice treated with vehicle or arsenic (0.5 or 5 ppm) via drinking water for six months. Scale bar, 20 μm.

To determine the effect of arsenic on p62 protein levels in keratinocytes, we first assessed whether arsenic alters p62 abundance in human HaCaT cells and in mouse skin. As compared with vehicle control, arsenic at 4 μM decreased p62 protein levels at earlier time points, while it increased the p62 protein level at 24 h (Figure 1A). At 8 μM, arsenic increased p62 protein levels at both 6 and 24 h (Figure 1A). The non-monotonic dose- and time-dependent response relationships are frequently reported in studies with endocrine disrupting chemicals including arsenic [25,26]. Meanwhile, it also increased Nrf2 protein abundance in a time-dependent manner preceding p62 up-regulation (Figure 1A), consistent with the known activation of Nrf2 by arsenic in HaCaT keratinocytes [8]. Furthermore, as compared with vehicle-treatment mice, p62 protein levels were increased in the epidermis of mice treated with arsenic (0.5 or 5 ppm) for six months (Figure 1B). These findings indicate that arsenic induces p62 up-regulation in vitro and in vivo.

### 2.2. Arsenic Induces p62 Expression Independent of Autophagy

**Figure 2:**
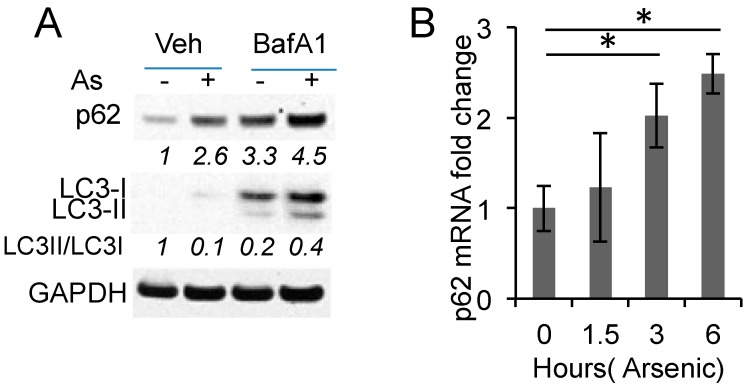
Arsenic induces p62 expression independent of autophagy: (**A**) (mmunoblot analysis of p62, LC3-I/II, and GAPDH in HaCaT cells treated with or without arsenic (8 μM) and bafilomycin A1 (BafA1, 10 nM) for 6 h. Cells were pretreated with BafA1 for 30 min prior to arsenic treatment; (**B**) Real-time PCR analysis of the p62 mRNA levels in HaCaT cells treated with or without arsenic (8 μM) over a time course. * *p* < 0.05, Student’s *t*-test, statistically significant between comparison groups.

To determine how arsenic up-regulates p62, we examined the role of autophagy. Arsenic increased p62 protein levels (Figure 2A). Inhibiting autophagic flux by the lysosome inhibitor bafilomycin A1 (BafA1) increased basal p62 protein levels, supporting p62 degradation by autophagy (Figure 2A). Arsenic treatment further increased p62 protein levels in BafA-treated cells, suggesting that arsenic induces p62 up-regulation at least in part through an additional mechanism. Indeed, arsenic increased the p62 mRNA levels in HaCaT cells (Figure 2B). These data indicate that arsenic induces p62 expression independent of autophagy.

### 2.3. Nrf2 Activation Is Required for Arsenic-Induced p62 Expression

**Figure 3:**
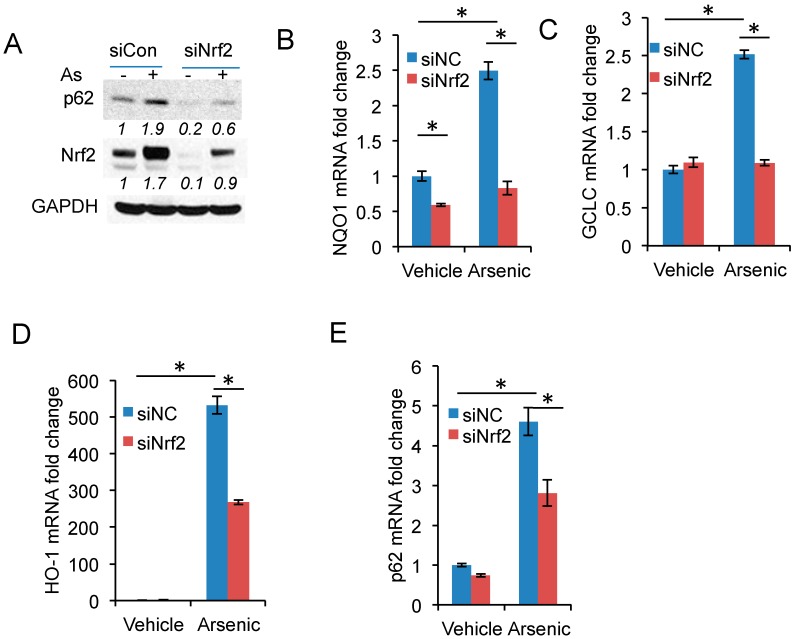
Nrf2 activation is required for arsenic-induced p62 expression: (**A**) Immunoblot analysis of p62, Nrf2, and GAPDH in HaCaT cells transfected with siRNA targeting negative control (siCon) or Nrf2 (siNrf2). The cells were treated with or without arsenic (8 μM) for 6 h; (**B**–**E**) Real-time PCR analysis of the mRNA levels of: NQO1 (**B**); GCLC (**C**); HO-1 (**D**); and p62 (**E**) in cells as in (**A**). * *p* < 0.05, Student’s *t*-test, statistically significant between comparison groups.

To determine the mechanism by which arsenic induced p62 expression, we assessed the role of Nrf2, a transcription factor regulating expression of multiple redox genes and the p62 gene [27,28]. Nrf2 knockdown reduced basal or arsenic-induced p62 protein levels or both (Figure 3A), as well as gene expression of known Nrf2 targets [27], including NQO1, GCLC, and HO-1 (Figure 3B–D). In addition, Nrf2 knockdown significantly reduced arsenic-induced p62 expression (Figure 3E).

### 2.4. p62 Inhibition Reduces Nrf2 Target Gene Expression

**Figure 4:**
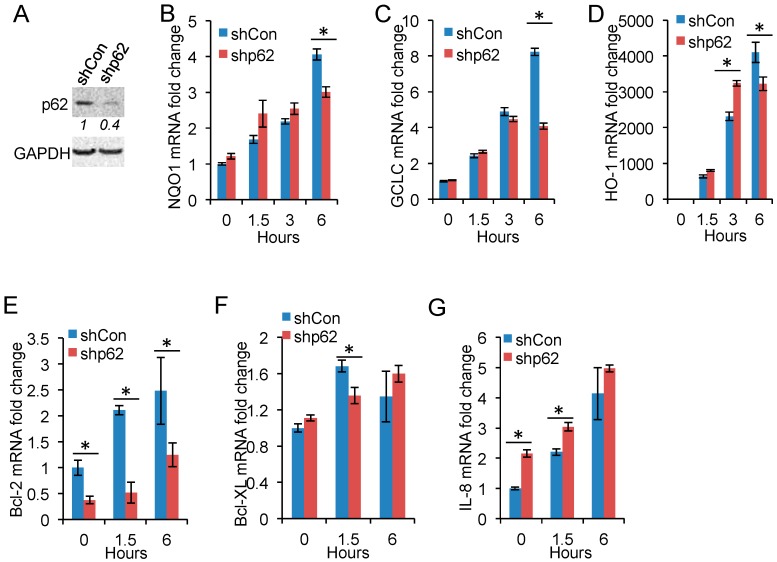
p62 inhibition reduces Nrf2 target gene expression: (**A**) immunoblot analysis of p62 and GAPDH in HaCaT cells stably infected with a lentiviral vector expressing shRNA targeting p62 (shp62) or a negative shRNA control (shCon); and (**B**–**G**) real-time PCR analysis of the mRNA levels of: NQO1 (**B**); GCLC (**C**); HO-1 (**D**); Bcl-2 (**E**); Bcl-XL (**F**); and IL-8 (**G**) in cells as in (**A**). The cells were treated with or without arsenic (8 μM) over a time course. * *p* < 0.05, Student’s *t*-test, statistically significant between comparison groups.

To determine the molecular function of arsenic-induced p62 expression, we assessed the effect of p62 knockdown on arsenic-induced gene expression of known targets of Nrf2 and NF-κB, since p62 is known to activate Nrf2 [28,29,30,31] and NF-κB [20]. p62 knockdown reduced arsenic-induced expression of NQO1, GCLC, and HO-1 (Figure 4A–D). In comparison, p62 knockdown decreased the expression of NF-κB target genes Bcl-2 and Bcl-XL (Figure 4E,F), while it increased the expression of IL-8 (Figure 4G). These data indicate that p62 is critical for Nrf2 activation but has a gene-specific effect on NF-κB activity.

### 2.5. p62 Up-Regulation Is Critical for Cell Proliferation Following Arsenic Treatment

**Figure 5:**
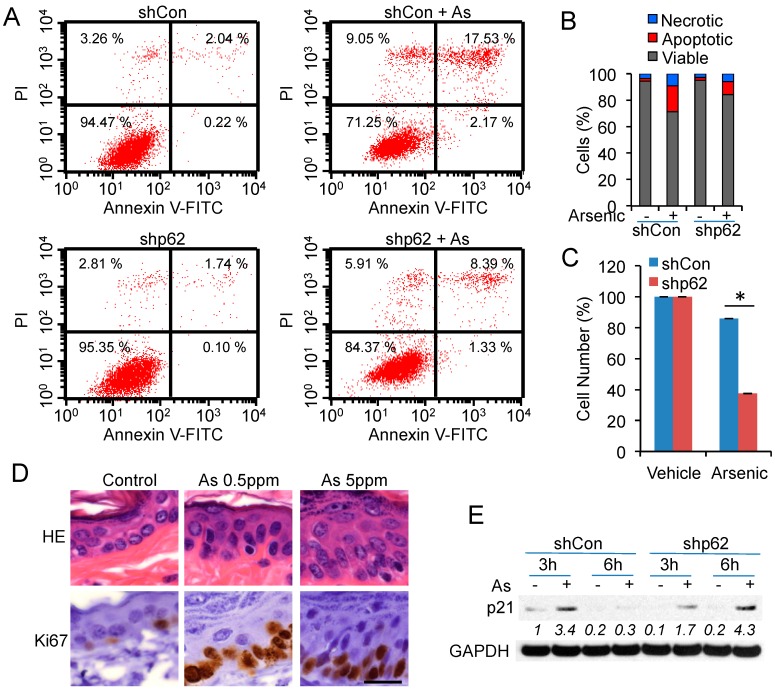
p62 up-regulation is critical for cell proliferation following arsenic treatment: (**A**) Flow cytometric analysis of apoptosis in shCon and shp62 cells treated with or without arsenic (25 μM) for 24 h; (**B**) Quantification of necrotic, apoptotic, or viable cells from (**A**); (**C**) CCK8 analysis of cell numbers in shCon or shp62 HaCaT cells treated with or without arsenic (20 μM) for 24 h. * *p* < 0.05, Student’s *t*-test, statistically significant between comparison groups; (**D**) Histological analysis after HE staining and immunohistochemical analysis of Ki67 in the epidermis of mice treated with or without arsenic (0.5 or 5 ppm). Scale bar, 20 μm; (**E**) Immunoblot analysis of p21 and GAPDH in shCon and shp62 HaCaT cells treated with or without arsenic (8 μM) for 3 or 6 h.

To determine the cellular function of arsenic-induced p62, we analyzed the effect of p62 knockdown on apoptosis and proliferation after arsenic exposure. Arsenic (25 μM) induced apoptosis and necrosis in HaCaT cells (Figure 5A,B). However, p62 knockdown did not significantly affect apoptosis induced by arsenic (Figure 5A,B). p62 knockdown did not affect basal cell proliferation, but significantly decreased cell proliferation after arsenic treatment (Figure 5C). Chronic arsenic exposure increased the number of Ki67-positive cells (Figure 5D), indicating an increase in cell proliferation in mouse epidermis, in association with p62 up-regulation (Figure 1B). In addition, enlargement of epidermal cell nuclei and prominent nucleoli were seen with higher dose arsenic treated tissues (Figure 5D), suggesting an early cell transformation towards precancerous lesion. Arsenic increased the protein levels of p21, a cell cycle inhibitor, at 3 h but not 6 h (Figure 5E). However, p62 knockdown induced p21 up-regulation at both 3 h and 6 h (Figure 5E). These results demonstrated that arsenic-induced p62 is critical for cell proliferation.

## 3. Discussion

Arsenic exposure is known to cause cancer in the skin. However, the underlying mechanism of arsenic carcinogenicity is poorly understood. Here we show that arsenic exposure induces p62 up-regulation in human HaCaT keratinocytes and mouse skin. Such p62 induction requires arsenic-induced Nrf2 activation. Interestingly, p62 up-regulation is crucial for arsenic-induced Nrf2 activity in association with cell proliferation. Our findings suggest a new molecular mechanism consisting of a p62/Nrf2 positive feedback loop in arsenic damage response and carcinogenicity.

Previous studies have demonstrated that arsenic leads to sustained Nrf2 activation, blockade of autophagic flux, and p62 up-regulation in different cell lines including 293T, iBMK, 3T3, BEAS-2B, and HBE cells [24]. p62 is a known substrate of autophagy for lysosomal degradation [13,14]. Therefore, a blockade of the autophagic flux can lead to p62 up-regulation following arsenic exposure. Arsenic-induced skin abnormalities as initial signs of arsenic poisoning and arsenic-induced skin cancers indicate skin tissue is a primary target of arsenic in humans [3]. Hence, we focused on the effect of arsenic in human epidermal keratinocytes and in mouse epidermis in comparison to the studies by Lau and colleagues in human bronchial epithelial cells [24]. Lau and colleagues demonstrated that arsenic caused inhibition of autophagic flux leads to induction of p62 (independent of Nrf2), further leading to sustained Nrf2 activation [24]. However, in human HaCaT keratinocytes, we found that arsenic induces autophagy and autophagic flux (Figure 2A), and arsenic induces p62 expression through Nrf2 activation (Figure 3) independent of autophagy (Figure 2B). We found that autophagic flux inhibition increases both basal and arsenic-induced p62 protein levels in HaCaT keratinocytes (Figure 2A). Clearly, human HaCaT keratinocytes activate a distinct mechanism to up-regulate p62 and Nrf2 activity independent of autophagy following arsenic exposure. Considering the potential oncogenic function of p62 in several human cancers [16,17,18,19,21] and a mouse lung tumorigenesis model [20], arsenic-induced p62 expression and up-regulation may contribute to arsenic carcinogenicity.

Indeed, we found that p62 is crucial for cell proliferation in response to arsenic. p62 knockdown reduced cell proliferation and led to sustained p21 up-regulation. In mouse epidermis, arsenic-induced p62 up-regulation was associated with increased proliferation. However, p62 had little effect in arsenic-induced apoptosis. At the molecular level, p62 is required for arsenic-induced Nrf2 activation, but had a gene-specific function in NF-κB activation. Previous reports have shown that p62 is known to activate Nrf2 [28,29,30,31] by interacting with the Nrf2-binding site on Keap1, a component of Cullin-3-type ubiquitin ligase for Nrf2, to compete with the interaction between Nrf2 and Keap1, resulting in stabilization of Nrf2 and transcriptional activation of Nrf2 target genes [29,30]. Following arsenic exposure, p62 and Nrf2 seem to form a positive feedback loop to foster Nrf2 activation in keratinocytes. As a transcription factor, Nrf2 protects cells and tissues from numerous toxicants and carcinogens through increasing the expression of a number of cytoprotective genes [32]. Specifically, Nrf2 was found to protect against arsenic-induced toxicity in vitro as well as in vivo [33,34,35,36]. Our data indicate that p62 is important for proliferation of human HaCaT keratinocytes after arsenic exposure, and suggest that arsenic-induced Nrf2, which is affected by p62, might promote proliferation for protecting keratinocytes against acute arsenic toxicity. Nrf2 has also been found to protect against carcinogenesis [37]. On the other hand, Nrf2 may also protect pre-cancerous or cancerous cells from chemotherapeutic agents and facilitate cancer progression. Nrf2 is aberrantly accumulated in many types of cancer, and its expression is associated with a poor prognosis in patients [32]. Therefore, p62/Nrf2 pathways may contribute to cell proliferation and carcinogenesis in response to arsenic in the skin.

## 4. Materials and Methods

### 4.1. Cell Culture and Arsenic Treatment

HaCaT cells were cultured as described previously [21]. Cells were treated with vehicle control or arsenic as described previously [8]. Arsenic concentrations of 4 and 8 μM were used since previous studies in keratinocytes and other cells indicated arsenic treatment in this dose range is relatively non-cytotoxic and induces signaling modulation consistent with arsenic induced skin carcinogenesis including oxidative stress and autophagy or p62 signaling [8,24,26,38].

### 4.2. Animals and Arsenic Treatment

All animals were handled according to the Guide for the Care and Use of Laboratory Animals, and studies have been approved by the University of Illinois at Chicago Institutional Animal Care and Use Committee. Male nude mice were purchased from Harlan at 4 weeks of Age. Mice were housed at 21 °C, on a 14:10-h light/dark schedule in polysulfone solid-bottom cages and double-deionized water was supplied from glass bottles. Animals were fed ad libitum a certified irradiated heavy metal-reduced diet (Teklad 2018C; Harlan). Nude mice (*n* = 3 per group) were treated with vehicle or sodium arsenic (0.5 or 5 ppm) via drinking water for six months. The 0.5 ppm concentration is consistent with environmentally relevant arsenic exposure associated with carcinogenesis in humans, including skin carcinogenesis [39,40,41]. A does of 5 ppm represents a higher dose of relevant arsenic exposure in humans and was used to verify the effect of arsenic in a dose–response manner compared to 0.5 ppm [42]. Mice were euthanized and skin samples from lower back were collected for immunohistochemical analysis of p62 as described previously [21].

### 4.3. siRNA and shRNA Transfection

HaCaT cells were transfected with negative control (siNC) or siRNA (ON-TARGETplus SMARTpool, Dharmacon, Lafayette, CO, USA) targeting Nrf2 using Amaxa Nucleofector (Lonza, Walkersville, MD, USA) according to the manufacturer’s instructions as described previously [43,44]. Knockdown of p62 in HaCaT cells was performed using lentiviral vectors as described previously [21]. Briefly, virus-containing supernatants were collected 24–48 h after transfection and used to infect recipients. Target cells were infected in the presence of Polybrene (8 μg/mL, Sigma–Aldrich, St. Louis, MO, USA) and selected with puromycin at 1 µg/mL for 6 days.

### 4.4. Western Blotting 

Protein concentration was determined using the BCA assay (Pierce, Rockford, IL, USA). Western blotting was performed as described previously [21,45]. Antibodies used included p62 (Progen Biotechnik GmbH, Heidelberg, Germany), LC3-I/II (Cell Signaling Technology, Danvers, MA, USA), GAPDH, Nrf2, and p21 (Santa Cruz Biotechnology, Santa Cruz, CA, USA).

### 4.5. Real-Time PCR

Quantitative real-time PCR assays were performed using a CFX Connect real-time system (Bio-Rad, Hercules, CA, USA) with Bio-Rad iQ SYBR Green Supermix as described previously [21,46]. Amplification primers are as follows. Human p62 gene, 5′-CAGAGAAGCCCATGGACAG-3′ (forward) and 5′-AGGTGCCTTGTACCCACATC-3′ (reverse); human GCLC gene, 5′-GATGCTGTCTTGCAGGGAATG-3′ (forward) and 5′-AGCGAGCTCCGTGCTGTT-3′ (reverse). Human HO-1 gene, 5′-GCCTGGAAGACACCCTAATGTG-3′ (forward) and 5′-GGCCGTGTCAACAAGGATACTT-3′ (reverse). Human NQO1 gene, 5′-TTCTGTGGCTTCCAAGTCTT-3′ (forward) and 5′-AGGCTGCTTGGAGCAAAATA-3′ (reverse). Human IL-8 gene, 5′-ATGACTTCCAAGCTGGCCGTGGCT-3′ (forward) and 5′-TCTCAGCCCTCTTCAAAAACTTCT-3′ (reverse). Human GAPDH gene, 5′-ACCACAGTCCATGCCATCAC-3′ (forward) and 5′-TCCACCACCCTGTTGCTGTA-3′ (reverse). Human BCL-XL gene, 5′-GACATCCCAGCTCCACATC-3′ (forward) and 5′-GTTCCCATAGAGTTCCACAAAAG-3′ (reverse). Human Bcl-2 gene, 5′-GTGGATGACTGAGTACCTGAAC-3′ (forward) and 5′-GCCAGGAGAAATCAAACAGAGG-3′ (reverse).

### 4.6. Determination of Apoptosis by Flow Cytometry

Apoptosis was determined by staining cells with Annexin-V/propidium iodide (PI) using Annexin V-FITC Apoptosis detection kit (Affymetrix eBioscience, San Diego, CA, USA) according to the manufacturer’s instructions (Affymetrix, Inc., Santa Clara, CA, USA). Cells positive for annexin V-fluorescein isothiocyanate (apoptotic cells) were quantified by flow cytometry using a BD Calibur (BD Biosciences, San Jose, CA, USA). Twenty-five-micromolar arsenic was used since similar concentrations have been indicated for arsenic-induced cytotoxicity in previous studies using in vitro models including keratinocytes [26,38,47].

### 4.7. Cell Viability Assay 

The numbers of cells were assessed with Cell Counting Kit-8 (CCK-8) (Sigma-Aldrich). The CCK-8 analysis was performed following the manufacturer’s protocol. Twenty-micromolar arsenic was used since similar concentrations have been indicated for arsenic’s effect on viability in previous studies using in vitro models including keratinocytes [26,38,47].

### 4.8. Histological and Immunohistochemical Analysis

Hematoxylin and eosin (H&E) staining of tissues and immunohistochemical analysis of p62 and Ki67 were performed by the Immunohistochemistry core facility at the University of Chicago.

### 4.9. Statistical Analyses

Statistical analyses were carried out using Prism 6 (GraphPad software, San Diego, CA, USA) as described previously [21]. Data are reported as mean ± S.E. Data were expressed as the mean of at least three independent experiments and analyzed by Student’s *t* test. A *p*-value of <0.05 was considered statistically significant. 

## 5. Conclusions

In conclusion, we found that, in human HaCaT keratinocytes, arsenic induces p62 expression through Nrf2 activation independent of autophagy. In mouse skin, chronic arsenic exposure increases p62 protein levels in association with increased cell proliferation in the epidermis. p62 knockdown reduces arsenic-induced Nrf2 activation and induced sustained p21 up-regulation. Our findings indicate that arsenic induces p62 expression to form a positive p62/Nrf2 feedback loop in arsenic response and suggest that targeting p62 may help prevent arsenic-induced skin cancer.
